# Valorisation of acid whey permeate for high-purity nisin Z production using artisanal *Lactococcus lactis* isolates

**DOI:** 10.1186/s12866-025-04543-x

**Published:** 2025-11-25

**Authors:** Humna Liaqat, Diana Paveljšek, Jernej Oberčkal, Bojana Bogovič Matijašić

**Affiliations:** Department of Animal Science, Biotechnical Faculty, University of Ljubljana, Groblje 3, Domžale, 1230 Slovenia

**Keywords:** Nisin Z, Bacteriocin, Acid whey permeate, *Lactococcus lactis*, RP-HPLC, Circular economy

## Abstract

The growing demand for sustainable bioprocessing has positioned whey, a side stream of the dairy industry, as a valuable substrate for producing high-value compounds. This study investigated the production and purification of nisin, a potent lantibiotic, using permeate (10 kDa cut-off) of ultrafiltered acid whey (PUFAW) devoid of lactoferrin as growth medium. Three strains of *Lactococcus lactis* subsp. *lactis*, including two artisanal isolates (IM 143 and IM 145) and a reference strain (IM 407), were evaluated for nisin production in PUFAW supplemented with yeast extract (YE) and mineral salts. Strain IM 145 showed superior growth and antimicrobial activity in PUFAW with YE (30 g/L) and produced up to 51,200 BA/mL of nisin. The bacteriocin was purified by ammonium sulphate precipitation followed by preparative RP-HPLC, yielding a product with 79.2% chromatographic purity. Tricine-SDS-PAGE and mass spectrometry confirmed the identity of the purified compound as nisin Z. This study presents a cost-effective, circular economy approach for the production of high-purity nisin from dairy side streams, with potential applications in food preservation and biomedicine.

**Supplementary Information:**

The online version contains supplementary material available at 10.1186/s12866-025-04543-x.

## Introduction

Acid whey is an important side stream of dairy industry, generated during the acid coagulation of milk by fermentation with lactic acid bacteria or by the addition of organic or mineral acids [1]. Its production has increased due to the growing demand for Greek yogurt, fresh curd cheeses, cottage cheese and related products [2]. While the disposal of acid whey is a major environmental problem and economically inefficient, its further processing is limited due to its high lactic acid and calcium content [3]. Nevertheless, acid whey and its fractions, such as ultrafiltration permeate, still contain sugars and nutrients that can be utilised by microorganisms. Some previous studies have focused not only on minimising the disposal costs of acid whey, but also on generating revenue from high value-added products [4]. In terms of sustainability, nisin production from dairy side-streams seems to be a good alternative, as nisin is recognised as safe (GRAS) by the US Food and Drug Administration (FDA) and listed as food additive E234 in the EU.

Nisin is the oldest known and most extensively studied bacteriocin, which is usually produced by lactococci and streptococci, and is active against several Gram-positive bacteria. Nisin belongs to the lantibiotic class of bacteriocins and exists in several variants, known as nisin A, Z, F, H, U, U2, Q, and others. Nisin A, Q, Z and F are commonly produced by *Lactococcus (L.) lactis*, nisin U and U2 by *Streptococcus uberis*, while others are produced by *Streptococcus*,* Staphylococcus*, or *Blautia* [5]. Nisin Z differs from nisin A by a single amino acid substitution – histidine is replaced at position 27 by asparagine which leads to a change in molecular weight from 3354 Da (nisin A) to 3331.05 Da (nisin Z) [6].

Nisin is currently used as a food preservative in dairy, vegetable and meat products. Its production is influenced by several factors, including fermentation conditions, growth medium composition, bacterial activity, growth phase, ionic strength, temperature, pH, and aeration. The biosynthesis of nisin reaches maximum concentration at the end of the exponential phase or at the beginning of the stationary phase [7].

Several studies have revealed that nisin can prevent the growth of multidrug-resistant microorganisms such as methicillin-resistant *Staphylococcus aureus*, *Streptococcus pneumoniae*, enterococci, and *Clostridioides difficile* [8]. In the past two decades, the use of nisin has been expanded to the biomedical field due to its natural antimicrobial and immunomodulatory effects [9]. However, its application in this field is limited by its low production yields and low purity level during fermentation.

Conventionally, nisin is produced in standard laboratory media such as MRS, M17, or BHI [10–14] and purified by various methods, including organic solvent extraction [15], immune-based techniques [14], chromatography [10, 11, 13, 16], salt precipitation [17], ammonium sulphate precipitation followed by chromatography, organic solvent extraction or hydrophobic interactions [12, 18]. Aqueous two-phase micellar systems have also been used [19].

In this study, permeate of ultrafiltered acid whey (devoid of lactoferrin) with various supplements was used for nisin production, requiring a specific purification approach involving ammonium sulphate precipitation followed by preparative reversed phase chromatography.

This strategy supports circular economy principles by valorising dairy industry side streams, reducing waste, and lowering the cost of natural food preservatives [20, 21]. The approach presented here enables the production of high-purity nisin suitable for both food and biomedical applications and represents a sustainable solution for the reuse of acid whey in line with zero-waste and circular economy goals.

## Methods

### Material

Acid whey produced during the manufacture of fresh curd cheese at the Celeia dairy (Slovenia) was initially processed on a large-scale by Arhel Ltd. (Slovenia) using microfiltration and monolithic chromatography to separate lactoferrin as a commercially valuable component [22]. The resulting flow-through fraction collected during chromatography, presented a side stream and was further ultrafiltered (10 kDa cut-off; Watersep Bioseparations Corp., USA) to remove most whey proteins. The resulting permeate (0.25 g/100 g protein, 3.81 g/100 g lactose, 0 g/100 g fat, and 5.26 g/100 g dry matter; pH 6.5) was used as the base for all growth media in our study. Nisin A and Z standards were obtained from Sigma (Missouri, USA) and Handary (Brussels, Belgium), respectively. All growth media and mineral salts - de Man-Rogosa and Sharpe (MRS) medium, Brain Heart Infusion (BHI) medium, M17 medium, and MgSO_4_, MnSO_4_, K_2_HPO_4_, CH_3_COONa, and CH_6_H_8_O_7_.2NH_3 −_ were purchased from Merck (Darmstadt, Germany). Yeast extract (YE) was purchased from Biolofe (Milan, Italy). All chemicals used for analysis were of analytical grade.

### Bacterial strains and growth conditions

Nisin was produced by three *L. lactis* subsp. *lactis* strains. Two strains, IM 143 (nisin Z producer, WGS available at https://www.ebi.ac.uk/ena/browser/view/CAKMBL010000000) and IM 145 (nisin Z producer, WGS available at https://www.ebi.ac.uk/ena/browser/view/CAKMAO010000000), were isolated from local Slovenian artisanal sour milk (isolated in 1995), and the reference strain IM 407, a nisin A producer, was purchased from ATCC (ATCC 11454). During initial screening of the isolates (1995), strains IM 143 and IM 145 were phenotypically characterised by phase contrast microscopy (cocci), Gram staining (Gram-positive), catalase test (negative), antimicrobial activity evaluation, and API 50 CH and biochemical tests API 20 Strep (Bio-Merieux, France) (results not shown). These two isolates, presumed to belong to the subspecies *L. lactis* subsp. *lactis*, were selected for further studies due to their broad-spectrum antimicrobial activity, which was reduced by trypsin.

All *Lactococcus* strains were grown in M17 or BHI media at 30 °C. *Latilactobacillus sakei* subsp. *sakei* IM 108 *(Lb. sakei)* - NCDO 2714 (National Collection of Dairy Organisms, National Institute for Dairying, Reading, England), used as an indicator strain, was grown in MRS medium and incubated anaerobically at 30 °C (Genbox, BioMérieux, France). All stock cultures were stored at −80 °C in M17 broth containing 20% (v/v) glycerol. Working cultures were kept in the same media at 4 °C.

### Nisin production

Five media were initially evaluated for production in 10 mL cultures: (1) BHI (commercial medium), (2) PUFAW, (3) PUFAW with 30 g/L YE, (4) PUFAW with 30 g/L YE and mineral salts (M1: 0.1 g/L MgSO_4_, 0.05 g/L MnSO_4_), and (5) PUFAW with 30 g/L YE and mineral salts (M2: 0.1 g/L MgSO_4_, 0.05 g/L MnSO_4_, 2 g/L K_2_HPO_4_, 2 g/L CH_3_COONa, 2 g/L CH_6_H_8_O_7_ × 2NH_3_). Strains were inoculated from overnight BHI cultures into each test medium. Cultures were incubated at 30 °C for 20 h. Optical density (OD) was measured at 630 nm in triplicate, with two technical replicates per sample. Antimicrobial activity was assessed using the agar diffusion test.

Following the initial screening, production was scaled up using a 2.5-L benchtop bioreactor (Bioengineering, Switzerland). For the 2-L production, the bioreactor was filled with 1820 mL PUFAW, 120 mL YE suspension of 50 g/100 mL (final YE concentration: 30 g/L), and 60 mL (3% v/v) of overnight lactococci culture. After temperature and pH adjustment (30 °C, pH 6.3), the medium was inoculated with strains IM 143, IM 145 or IM 407, and the vessel was flushed with nitrogen for 15 min. The bioprocess was carried out for 24 h at 200 rpm. Growth was monitored by measuring the optical density (OD). After the process, the pH was lowered to 3.0 using 37% (v/v) HCl (Merck, Darmstadt, Germany), and bacterial cells were removed by centrifugation at 12,000× g for 30 min at 4 °C. The resulting cell-free supernatant (CFS) was filtered through 0.45 μm membrane filters and analysed for nisin activity using agar diffusion and microdilution assays. The CFS was further subjected to ammonium sulphate precipitation and RP-HPLC for purification.

### Agar diffusion assay

To detect antimicrobial activity, overnight cultures of the indicator strain *Lb. sakei* were added onto soft MRS agar (7.5 g/L agar-agar) at a final concentration of 10⁵–10⁶ CFU/mL. The agar was poured into Petri dishes (15 mL per plate). Each CFS sample (5 µL) was spotted on the agar surface, dried, and incubated anaerobically at 30 °C for 16–18 h. Inhibition zones indicated antimicrobial activity.

### Microdilution assay

The antimicrobial activity of CFS and other fractions obtained during purification was measured by a critical dilution assay using *Lb. sakei* NCDO 2714. Antimicrobial activity was expressed in arbitrary units (BA/mL, bacteriocin activity per mL) [23]. In 96-well plates, 50 µL of serially diluted samples were mixed with 150 µL of the indicator strain culture (10⁴ CFU/well). Plates were incubated anaerobically for 16–20 h at 30 °C. Inhibition was measured at 630 nm using a BioTek Cytation 3 plate reader. BA/mL was calculated based on the lowest dilution resulting in ≥ 50% inhibition compared to the MRS control.

### Purification of Nisin

#### Precipitation with ammonium sulphate

A saturated solution of ammonium sulphate (685 g/L) was prepared in Milli-Q water and stirred at 40 °C. The solution was mixed with the CFS in a 2:3 ratio (1000:1500 mL) and stirred overnight at 4 °C. After centrifugation at 12,000× g for 15 min at 4 °C, the pellet was dissolved in 100 mL citrate buffer (10 mM citric acid (C₆H₈O₇), pH 3), filtered, and stored at −20 °C. Antimicrobial activity was monitored at each step by agar diffusion and microdilution assays.

The nisin Z purification yield was calculated as:

$$\begin{aligned} &\lbrack(\mathrm{BA}/\mathrm{mL})\times\mathrm{V_f}(\mathrm{mL})/(\mathrm{BA}/\mathrm{mL})\times\mathrm{V_{CFS}}(\mathrm{mL})\rbrack\times100,\\&\mathrm{where}\;{\mathrm V}_{\mathrm f}\;\mathrm{is}\;\mathrm{the}\;\mathrm{volume}\;\mathrm{of}\;\mathrm{the}\;\mathrm{purified}\;\mathrm{fraction}\;\\&\mathrm{and}\;{\mathrm V}_{\mathrm{CFS}}\;\mathrm{is}\;\mathrm{the}\;\mathrm{volume}\;\mathrm{of}\;\mathrm{the}\;\mathrm{initial}\;\mathrm{CFS}.\end{aligned}$$

#### Preparative RP-HPLC

The resuspended pellet (in citrate buffer) was mixed 1:1 with mobile phase A and filtered through a 0.22 μm filter before being loaded onto a Jupiter 4u Proteo 90 A 250 × 10.0 mm column (Phenomenex). Proteins were eluted using a binary gradient: 0.1% trifluoroacetic acid (TFA) and 5% acetonitrile (ACN) in Milli-Q water as mobile phase A, and 0.1% TFA and 95% ACN in Milli-Q water as mobile phase B. The following gradient profile was used: 0–2 min, 0 to 20% B; 2–32 min, 20 to 50% B; 32–34 min, 50 to 100% B; 34–39 min, 100% B; 39–41 min, 100 to 0% B; 41–51 min, 0% B. The flow rate was 4 mL/min and peaks were detected at 214 nm. Fractions showing antimicrobial activity were re-chromatographed for higher purity and analysed by sodium dodecyl sulphate-polyacrylamide gel electrophoresis (Tricine-SDS-PAGE), analytical RP-HPLC, and mass spectrometry.

#### Tricine SDS–PAGE

Organic solvents were removed from samples using a MiVac vacuum concentrator (Genevac, United States). Pellets were resuspended in 10 mM citrate buffer (in the same volume as the starting solution). The nisin standard (2 mg/mL) was also dissolved in citrate buffer. All other samples were analysed as received. The nisin standard and RP-HPLC fractions were subjected to Tricine-SDS-PAGE (Bio-Rad Laboratories, Hercules, CA, USA) as described in the literature [24, 25]. Samples and standards were dissolved in a non-reducing loading buffer (1:1), mixed by vortexing, heated at 95 °C for 5 min, cooled, and centrifuged at 14,000 × g. To each well, 3 µL (1 mg/mL) of the nisin standard and 14 µL of the HPLC fractions were added. A discontinuous Tris–Tricine gel system was used (stacking: 4%; spacer: 10%; resolving: 15% polyacrylamide), containing 0.1% SDS. Electrophoresis was performed at 80 mA until samples entered the gel, then at 120 mA. Gels were stained with 0.25% Coomassie Brilliant Blue R250 and destained in 30% ethanol/10% acetic acid.

#### Analytical RP-HPLC

Analytical RP-HPLC was performed on a Shimadzu HPLC system (Shimadzu, Kyoto, Japan) with a diode array detector and a Phenomenex C8 Widepore Aeris column (3.6 μm, 200 Å, 250 × 4.6 mm) heated to 45° C. Spectrophotometric detection was performed in the 190–600 nm range. The column was eluted with a binary gradient using HPLC solvents: (A) 0.1% TFA in Milli-Q water and (B) 0.1% TFA in ACN.

Samples and standards were mixed 1:1 with mobile phase A. Up to 40 µL of a Nisin Z standard (5 mg/mL) and pooled fractions were injected at a flow rate of 1 mL/min. The elution programme was as follows: a linear gradient from 20% to 38% B from 0 to 18 min, an increase to 100% B in 2 min, a wash with 100% B for 4 min, a decrease to 20% B in 1 min and finally re-equilibration of the column with 20% B for 5 min.

Chromatograms were analysed using LabSolutions LC/GC 5.32 SP1 (Shimadzu, Nakagyo-Ku, Kyoto, Japan). Peaks with retention times corresponding to those of the two standards were integrated and the nisin concentration was determined using a calibration curve from standards (1–15 µL, 5 mg/mL).

#### Mass spectrometry

Selected RP-HPLC fractions containing the purest proteins corresponding to the nisin peak were analysed using a Q-Tof Premier high-resolution tandem mass spectrometer (Micromass, Waters, Manchester, UK). An electrospray ionisation (ESI) source was used to ionise the protein solution, followed by injection into the mobile phase consisting of water with 0.1% formic acid and acetonitrile (50:50) at a flow rate of 10 µL/min. A cone voltage of 40 V and a capillary voltage of 3 kV in positive ion mode (ESI+) were used. The desolvation gas nitrogen at 3500 °C and the source temperature of 1200 °C were used to create optimal spraying conditions in the ion source. Mass spectra were acquired in continuum mode over an m/z range of 500 to 3000, with a scan time of 1 s and an inter-scan time of 0.1 s, at a mass resolution of 10,000 for accurate high-resolution mass measurements. The transformed mass spectra of the proteins were calculated using Maximum Entropy software.

### Statistical analysis

Antibacterial activity tests in microtiter plates were performed in triplicate. Two-way ANOVA with Dunnett’s multiple comparison was used to compare growth (OD) in PUFAW media with and without supplements (GraphPad Prism 8.4.3). The significance of differences in antimicrobial activity of the CFSs at different time points during the fermentation process in the bioreactor was assessed by the Kruskal-Wallis test with Dunnett’s multiple comparisons, with differences considered statistically significant at *p* < 0.05. Error bars represent standard errors of the mean, calculated with SigmaPlot 11.0 (Erkrath, Germany).

## Results

### Nisin production

We first compared the growth and nisin production of three *Lactococcus lactis* subsp. *lactis* strains in various media on a small scale (10 mL): PUFAW, PUFAW supplemented with 60 g/L yeast extract (YE), PUFAW supplemented with 60 g/L YE and with one of the mineral salt mixtures (M1 or M2) and BHI.

**Fig. 1:**
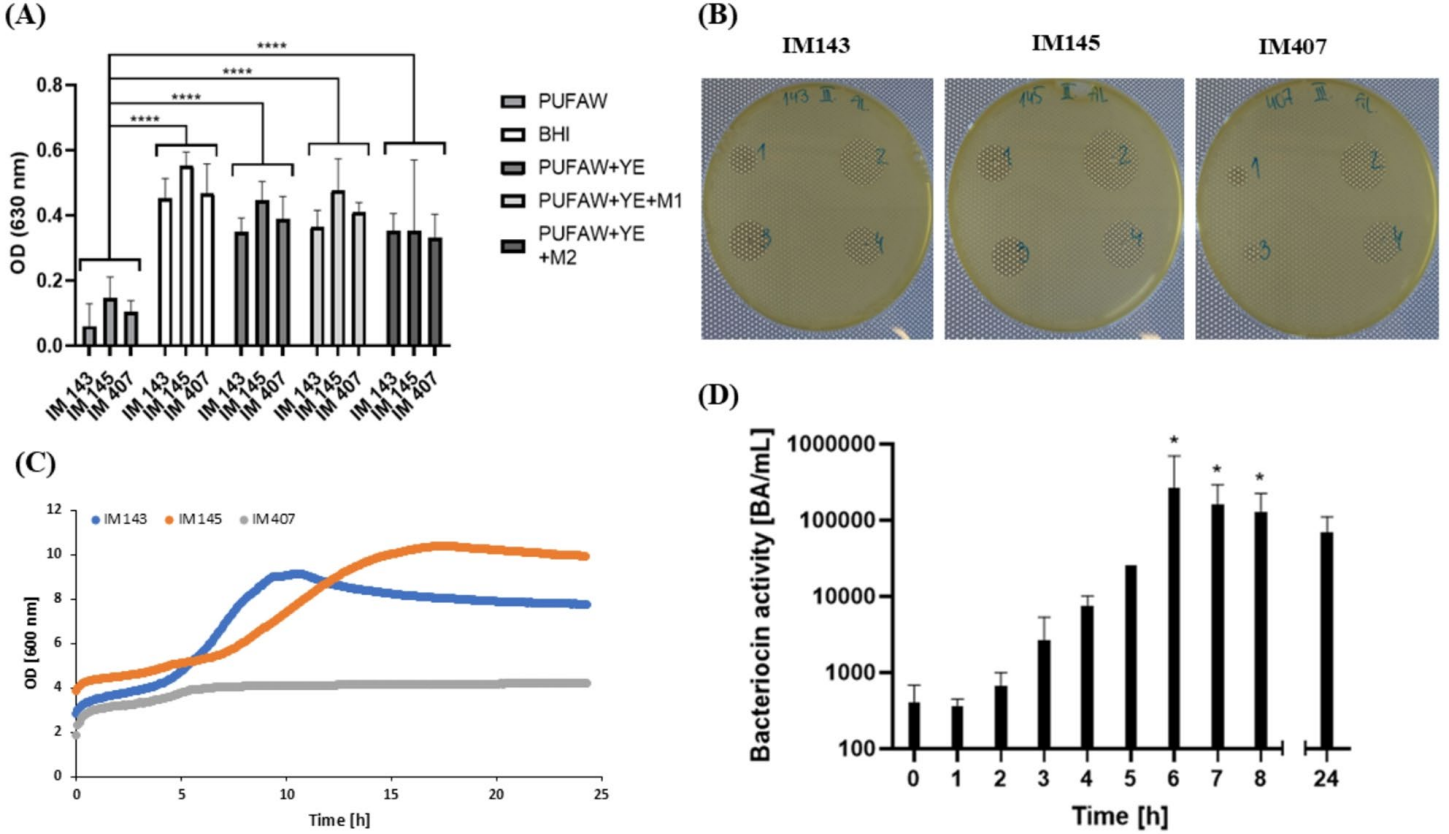
Comparison of different culture media for the cultivation of *Lactococcus lactis* strains and antimicrobial activity. **A** Growth of three strains in different culture media (10 mL), measured after 20 h. BHI, brain-heart infusion broth; PUFAW, permeate of ultrafiltered acid whey; YE, yeast extract; M1, mixture of mineral salts (MgSO_4_, MnSO_4_); M2, mixture of mineral salts (MgSO_4_, MnSO_4_, K_2_HPO_4_, CH_3_COONa, CH_6_H_8_O_7_.2NH_3_). Results are presented as the mean of three measurements ± 95% confidence interval. The asterisk (****) indicates astatistically significant difference in growth (OD) in PUFAW media with supplements compared to PUFAW media without supplements (two way ANOVA with Dunnett’s multiple comparisons, *p* < 0.0001). **B** Antimicrobial activity in the cell-free supernatants of three nisin-producing cultures using different culture media. 1, PUFAW; 2, PUFAW + YE; 3, PUFAW + YE + M1; 4, PUFAW + YE + M2. **C** Growth curves of three nisin-producing strains in PUFAW supplemented with YE (2 L, pH 6.3). **D** Antimicrobial activity of IM 145 culture supernatant in PUFAW + YE at different time points during the fermentation process in the bioreactor (2 L). Results are presented as the mean of three measurements ± 95% confidence interval. * - Statistically significant difference compared to the initial time point (Kruskal-Wallis test with Dunn’s multiple comparisons, *p* < 0.05)

PUFAW alone supported the least growth across all strains. The addition of YE significantly enhanced growth (*p* < 0.0001), whereas mineral salt supplementation (M1 or M2) did not result in further improvement (Fig. 1A). Two-way ANOVA confirmed significant effects of both strain (*p* = 0.0268) and medium (*p* < 0.0001). As Tukey’s post hoc comparison did not reveal significant differences between strains within the same medium, only Dunnett’s comparisons to PUFAW are presented in Fig. 1 (A).

Antimicrobial activity in the cell-free supernatants (CFSs) after 18 h of culture was evaluated using the agar diffusion assay (Fig. 1B). All strains produced bacteriocins (presumably nisin) in all tested media, as evidenced by growth inhibition zones against the indicator strain. Isolates IM 143 and IM 145 showed larger inhibition zones than the reference strain (IM 407), and antimicrobial activity was notably higher in PUFAW supplemented with YE.

Next, nisin production was scaled up using a 2-L bioreactor with PUFAW + YE as the growth medium. Growth curves showed that IM 145 reached the highest OD₆₃₀, while IM 407 exhibited limited growth and the lowest specific growth rate (µₘₐₓ = 0.078 h⁻¹) (Fig. (C)). IM 143 had the fastest growth (µₘₐₓ = 0.188 h⁻¹), but IM 145 produced the most bacteriocins, reaching 51,200 BA/mL after 24 h compared to 25,600 BA/mL from IM 143. Thus, IM 145 was selected for further experiments.

For strain IM 145, which reached the highest antimicrobial activity at the end of the process, antimicrobial activity was monitored hourly. Time-course analysis showed that antimicrobial activity in the culture supernatant increased, reaching its peak after 6 h, followed by a slight decline up to 24 h (Fig. 1D).

### Isolation of Nisin

In the initial purification step, nisin present in the supernatant of the *L. lactis* IM 145 culture grown in PUFAW medium was concentrated by ammonium sulphate precipitation. The initial antimicrobial activity in the fermentation broth (pH 6.3) was 32,000 BA/mL. After adjusting the pH to 3 and removing bacterial cells, activity increased to 64,000 BA/mL. The resuspended pellet (100 mL) exhibited 512,000 BA/mL, corresponding to a 40% yield.

No precipitation was observed when the resuspended pellet from the ammonium sulphate precipitation was mixed with mobile phase A prior to preparative RP-HPLC, and all antimicrobial activity was retained by the column, as verified in the flow-through fraction. Antimicrobial activity was measured in each fraction, and the most active fractions were re-chromatographed for enhanced purity. Final fractions showing antimicrobial activity were analysed by Tricine-SDS-PAGE and analytical RP-HPLC.

**Fig. 2:**
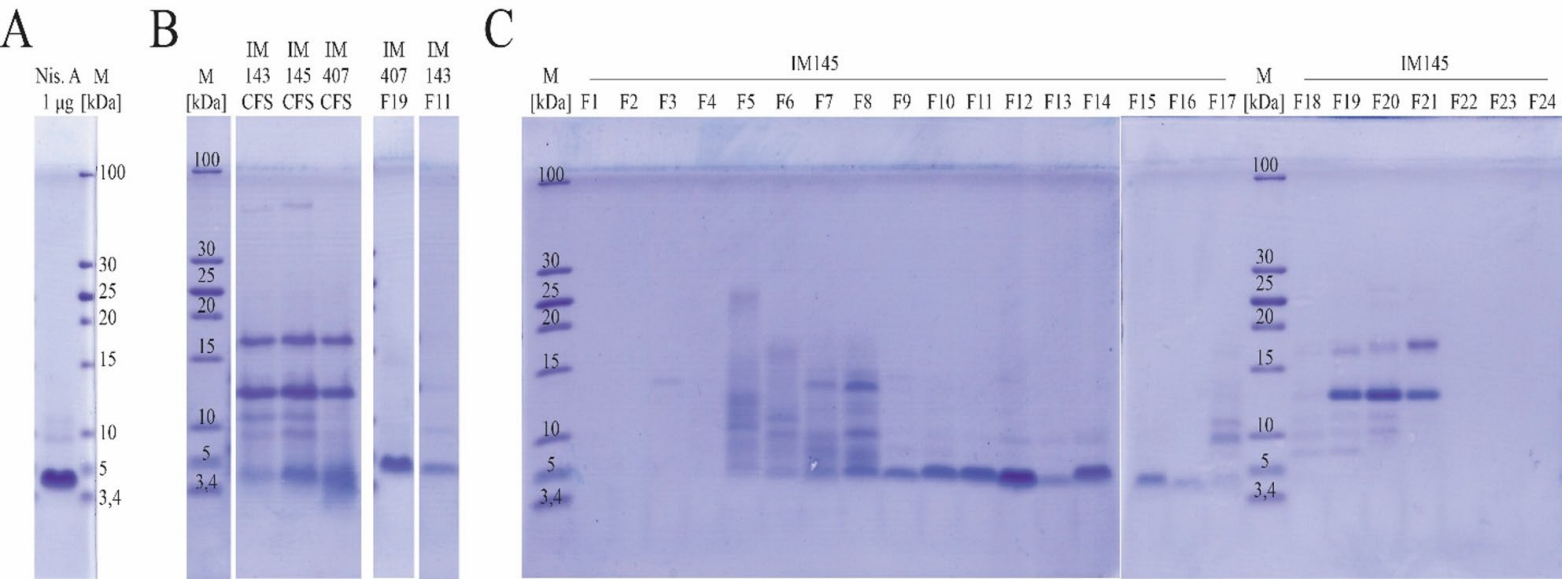
Detection of nisin produced by the strains *L. lactis* subsp. *lactis* IM 143, IM 145, and IM 407. **A** Nisin A standard (reference band). **B** The cell-free supernatant (CFS) of cultures IM 143, IM 145, and IM 407 grown in PUFAW + YE, and two fractions from IM 143 and IM 407 with the highest antimicrobial activity, eluted during HPLC and sent for LC-MS analysis. **C** All fractions of IM 145 eluted during HPLC

Tricine-SDS-PAGE revealed a band at approximately 3.5–5 kDa—corresponding to nisin—in the nisin A standard (Fig. 2A), in the CFS of all three strains (Fig. 2B), in fractions from IM 143 and IM 407 with the highest antimicrobial activity (Fig. 2B), and in several fractions from IM 145 (Fig. 2C). Slight variations in band position may reflect differences in molecular structure. Additional prominent bands between 10 and 20 kDa were likely residual whey proteins such as α-lactalbumin and β-lactoglobulin. Based on antimicrobial activity and Tricine-SDS-PAGE, fractions F11 (IM 143), F11 (IM 145) and F19 (IM 407) were selected for LC-MS analysis.

### Identification of Nisin isolates by RP-HPLC and mass spectrometry

**Fig. 3:**
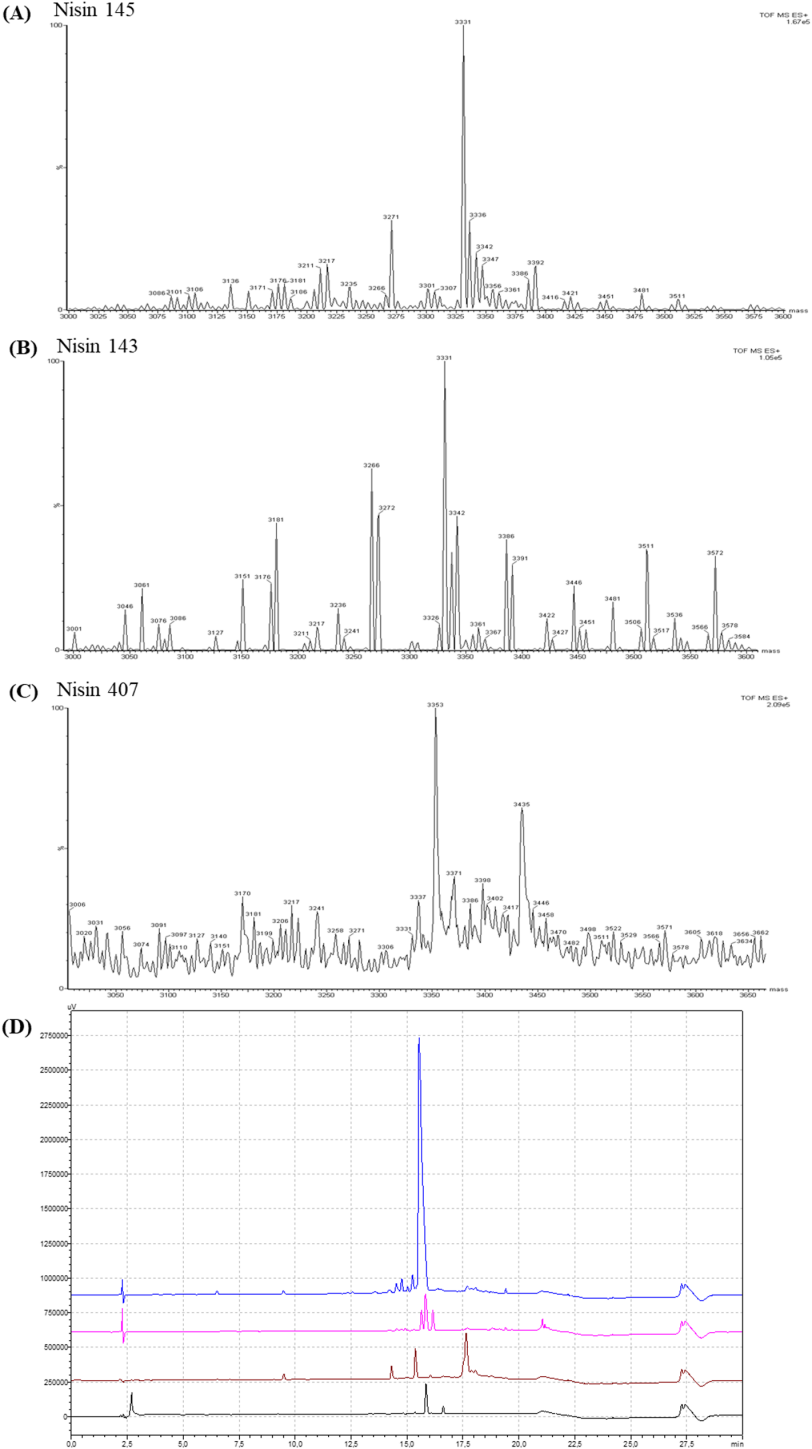
RP-HPLC chromatogram (**D**) and mass spectra (**A**, **B**, **C**) of selected nisin-containing fractions collected during preparative RP-HPLC. The black line in the chromatogram (D) represents the nisin Z standard, while pink, blue, and brown lines represent the fractions of the strains *L. lactis* subsp. *lactis* IM 143, IM 145 and IM 407, respectively. The molecular masses of the highest peaks in the mass spectra of samples IM 143 and IM 145 corresponded to those of nisin Z, and the molecular mass of the highest peak of the reference strain IM 407 (nisin A producer) corresponded to that of nisin A

Analytical RP-HPLC showed that the main peaks in the chromatograms of strains IM 143 and IM 145 (pink and blue lines, respectively) had the same retention time as the nisin Z standard (black line) Fig. 3(D). The peak of the IM 407 sample (brown line) eluted differently and likely corresponded to nisin A, consistent with its known production profile [26]. Purified nisin from IM 145 exhibited a chromatographic purity of 79.2% (area percentage), which exceeded that of the commercial nisin Z standard (62.3% pure). The purities of nisin from IM 143 and IM 407 were 34.4% and 43.3%, respectively. As multiple peaks were present, we determined the purity of the peak most likely representing nisin. Due to solubility issues with the commercial nisin A in mobile phase A, the purity of nisin from IM 143 could not be determined with certainty. The major peak at 17.7 min was presumed to represent nisin A, as it matched the dominant mass in MS analysis.

Mass spectrometry analysis confirmed the identity of the isolated nisins. The IM 145 and IM 143 samples both showed a dominant peak at 3331 Da, consistent with the molecular weight of nisin Z (3331.1 Da) (Fig. 3(A), 3(B)). The IM 407 sample showed a peak at 3353 Da, which is close to the molecular weight of nisin A (3354.07 Da) (Fig. 3C). All spectra included multiple molecular ions, indicating the presence of nisin variants or possible oligomers.

## Discussion

This study demonstrates the feasibility of using ultrafiltered acid whey permeate (PUFAW) as a sustainable and cost-effective medium for the production of nisin, a potent lantibiotic.

While previous studies have shown nisin production using various whey fractions or semi-products from whey, including sweet whey, acid whey, whey protein concentrate (WPC), whey permeate, and soy whey [2, 16, 17, 26–32], this study is distinct in its use of the permeate of ultrafiltered acid whey, which remains after the separation of whey proteins from acid whey and is commonly treated as waste. In addition, we used unique isolates from artisanal fermented milk, established a preparative-scale protocol for nisin Z purification, and confirmed the identity of nisin by mass spectrometry.

It is well established that nisin biosynthesis in *L. lactis* is strongly influenced by environmental and nutritional factors, including pH, aeration, temperature, and the composition of the growth medium. Optimising nisin yield often requires the addition of nutrients such as yeast extract, sucrose, soybean peptone, and mineral salts, as well as careful control of fermentation parameters such as pH and feeding strategies [16, 17, 19, 26–34]. In line with these findings, we observed that growth and nisin production by all tested *Lactococcus lactis* subsp. *lactis* strains - IM 143, IM 145, and the reference strain IM 407 - were strongly enhanced by YE supplementation (30 g/L). YE is rich in amino acids, peptides, and vitamins, which are critical for bacteriocin biosynthesis and bacterial growth. The lack of additional benefit from mineral salt supplementation is not unexpected, given that whey already contains a variety of minerals [35, 36]. Further improvements in nisin production might be achieved by including other nitrogen sources, using continuous fermentation, controlled feeding with substrates such as sugars, the addition of nisin as an inducer, or the use of immobilised bacterial cells [27–29, 37].

Whey composition and properties vary with cheese manufacturing practices, including pH, temperature, and starter cultures used. A closely related study by Xia et al. (2005) used the permeate of ultrafiltration of sweet whey from Cheddar cheese production, supplemented with yeast extract or casein hydrolysate (5–40 g/L) and Tween 80 (0–5 g/L), and demonstrated successful nisin production in continuous fermentation with immobilised *L. lactis* subsp. *lactis* ATCC 11,454 [29]. Similarly, Desjardins et al. (2001) used 6% reconstituted commercial dried sweet whey permeate with 10 g/L yeast extract and 0.1% Tween 80. Nisin Z was produced by *L. lactis* subsp. *lactis* biovar *diacetylactis* UL719 in both continuous free-cell and immobilised-cell systems, although yields were not higher than those achieved in batch cultures [32].

Among the strains tested in our study, IM 145 exhibited the highest bacteriocin production, reaching 51,200 BA/mL. Notably, antimicrobial activity peaked after 6 h and then declined slightly, consistent with reports that nisin production typically reaches its maximum during the late exponential or early stationary growth phases [7, 34]. The subsequent decline could be attributed to adsorption of nisin onto bacterial cells or its proteolytic degradation [30]. However, direct comparison of the amounts of nisin produced in whey-based media across different studies is difficult due to methodological differences. Forexample, Xia et al. (2005) used the agar spot test with *Lactobacillus leichmannii* ATCC 4797 as the indicator strain and expressed nisin activity in arbitrary units per mL, per gram of dry cell weight, or per gram of lactose consumed [29]. Various indicator strains have been used in biological tests for determination of antimicrobial activity of nisin, including *Bacillus luteus* [12, 26], *L. lactis* subsp. *cremoris* [17], *Weissella paramesenteroides* ATCC 33313 [13]), *Pediococcus acidilactici* UL5 [32], *Streptococcus agalactiae* [18], *Lb. sakei* ATCC 15521 [19], *Carnobacterium maltaromaticum* (formerly *Carnobacterium piscicola*) CECT 4020 [31], *Lb. sakei* ATCC 15521 [16], nisin-susceptible *L. lactis* NZ9000 [11] and *Lb. sakei* NCDO 2714 (present study). Assay formats also vary: agar well diffusion, agar spot diffusion, critical dilution assay in tubes [31], critical dilution in microtiter plates [11, this study] where 50% inhibition is observed, or the highest dilution showing inhibition is considered [18], or diameters of inhibition zones are measured [16, 30]. Nisin activity is typically reported in arbitrary units (AU), making cross-study comparisons imprecise. An improved approach involves using commercial nisin standards of known concentration (µg or IU per gram), and constructing standard curves to convert AU into µg or IU [17, 19, 32, 33], based on the accepted equivalence of 40 IU per 1 µg of pure nisin A.

The lack of standardisation in bacteriocin activity measurement also complicates the evaluation of purification efficiency. Yields may be reported as recovery percentages based on AU or IU per volume or protein content, or as ratios of nisin to total protein before and afterpurification. In this study, ammonium sulphate precipitation resulted in a 40% recovery of antimicrobial activity. This is lower than reported by Tafreshi (2020), who achieved 90% yield (IU-based), and Gujarathi et al. (2008), who reported 62% (AU-based), though their production media (MRS broth) differ significantly from PUFAW [12, 18].

In the reverse-phase chromatography condusted in this study, only the purest and most active fractions were collected and rechromatographed to obtain nisin suitable for further biological activity testing on cell cultures.Consequently, the overall recovery yield could not be calculated, but chromatographic purity was determined. The final nisin Z product from IM 145 achieved 79.2% purity, surpassing both the commercial nisin Z standard (62.3%) and previous reports such as Prioult et al. (60%) and Gough et al. (45.7%) using whey permeate-based media [14, 17].

In Tricine SDS-PAGE analysis, minor shifts in band migration were observed, which may reflect differences in molecular structure, charge, or conformation [38]. Nisin was not detected in fractions dominated by whey proteins such as α-lactalbumin and β-lactoglobulin (Fig. 2). In earlier RP-HPLC trials with a C4 column, nisin eluted across multiple whey-protein-rich fractions, complicating isolation. This issue was resolved by using a C12 column. Multiple peaks observed in the preparative RP-HPLC chromatograms likely correspond to different molecular variants or oligomeric forms of nisin. High concentrations of acetonitrile (ACN) and trifluoroacetic acid (TFA) in the mobile phase may also promote aggregation or alter retention.

Mass spectrometry revealed multiple molecular ions in all samples, supporting the presence of different nisin forms. RP-HPLC analysis and MS confirmed that IM 143 and IM 145 produced nisin Z, as expected from their genetic backgrounds. The nisin Z gene was previously identified in the genomes of IM 143 and IM 145, which were sequenced in the study of Rozman et al. (2023) (https://www.ebi.ac.uk/ena/browser/view/CAKMAO010000000) [39]. Protein sequence alignment using BLASTp confirmed the presence of *nisZ* in the genomes of *L. lactis* subsp. *lactis* IM 143 and IM 145 (Supplementary Fig. 1) [40].

The use of acid whey permeate for nisin Z production offers economic benefits by valorising a dairy by-product and generating a high-value compound. This approach aligns with circular economy principles, promoting sustainability and resource efficiency in the dairy industry, which has attracted increasing attention in recent years [3, 20, 41]. Recent studies show that valorising whey and whey permeate is both economically viable and scalable, making it a key strategy for advancing circular economy practices in this sector. Scalable technologies have been developed to convert these streams into value-added products such as bioethanol, lactic acid, bacteriocins, biogas, and bioplastics [3, 4, 20, 21, 28, 29, 42]. Advances in fermentation, biorefinery integration, and membrane technologies have further improved yields and reduced production costs, enhancing industrial feasibility.

For example, producing the bacteriocin sakacin A from cheese whey permeate supplemented with meat and yeast extracts significantly lowers manufacturing costs and expands its potential applications as an anti-*Listeria* agent [20]. Similarly, a novel techno-economic approach using genetically engineered lactic acid bacteria and process intensification has enabled efficient conversion of whey permeate into D-lactic acid, providing both environmental benefits and revenue opportunities for the dairy industry [42]. A similar strategy could be applied to develop bacteriocin-based products.

Innovative, cost-effective, and scalable technologies are emerging, integrating anaerobic digestion, biomass production, and downstream processing into biofuels, pigments, antioxidants, and antimicrobial agents such as bacteriocins [43, 44]. Coupling nisin Z production from acid whey permeate with such innovations represents a promising sustainable waste management strategy that aligns with both environmental and economic objectives in the dairy sector.

## Conclusion

This study presents a sustainable and cost-effective approach for producing high-purity nisin using permeate of ultrafiltered acid whey (PUFAW), a dairy industry side stream. Of the three tested *Lactococcus lactis* subsp. *lactis* strains, isolate IM 145 showed the highest nisin production when grown in PUFAW supplemented with yeast extract. The bacteriocin was purified using ammonium sulphate precipitation followed by preparative RP-HPLC, yielding nisin Z with 79.2% chromatographic purity.

The identity of the purified product was confirmed by Tricine-SDS-PAGE, analytical RP-HPLC, and mass spectrometry. These results demonstrate the potential of PUFAW as a fermentation substrate within a circular economy framework, supporting waste valorisation and the sustainable production of natural antimicrobials.

## Supplementary Information

Supplementary Material 1.

Supplementary Material 2.

## Data Availability

The datasets generated and/or analysed during the current study are available in the Mendelay Data repository, doi: 10.17632/565sknfykx.1. https://data.mendeley.com/preview/565sknfykx?a=4ed1446f-4f6b-43a1-9066-535b814d719d.
