# Supplementary material for: Valorisation of acid whey permeate for high-purity nisin Z production using artisanal Lactococcus lactis isolates
Source: BMC Microbiol. 2025 Nov 25;26:9. doi: 10.1186/s12866-025-04543-x (PMC12777345; doi:10.1186/s12866-025-04543-x)
Supplement: Supplementary file 1 — Supplementary Material 1. [file 12866_2025_4543_MOESM1_ESM.docx]

IM145 MSTKDFNLDLVSVSKKDSGASPRITSISLCTPGCKTGALMGCNMKTATCNCSIHVSK* 57

IM143 MSTKDFNLDLVSVSKKDSGASPRITSISLCTPGCKTGALMGCNMKTATCNCSIHVSK* 57

CAA43440.1 MSTKDFNLDLVSVSKKDSGASPRITSISLCTPGCKTGALMGCNMKTATCNCSIHVSK* 57 ***************************************************************************

**Supplementary Fig. 1. Protein BLASTp alignment showing that the strains LM143 and IM145 produce nizin Z. Nisin A has H instead of N t the site indicated with underlined letter.**

Sequence CAA43440.1: https://pubmed.ncbi.nlm.nih.gov/1935953, Mulders 1991

Reference:

Mulders JW, Boerrigter IJ, Rollema HS, Siezen RJ, de Vos WM (1991) Identification and characterization of the lantibiotic nisin Z, a natural nisin variant. Eur J Biochem 201 (3):581-584. doi:10.1111/j.1432-1033.1991.tb16317.x
