# Supplementary figures and images for: Valorisation of acid whey permeate for high-purity nisin Z production using artisanal Lactococcus lactis isolates
Source: BMC Microbiol. 2025 Nov 25;26:9. doi: 10.1186/s12866-025-04543-x (PMC12777345; doi:10.1186/s12866-025-04543-x)

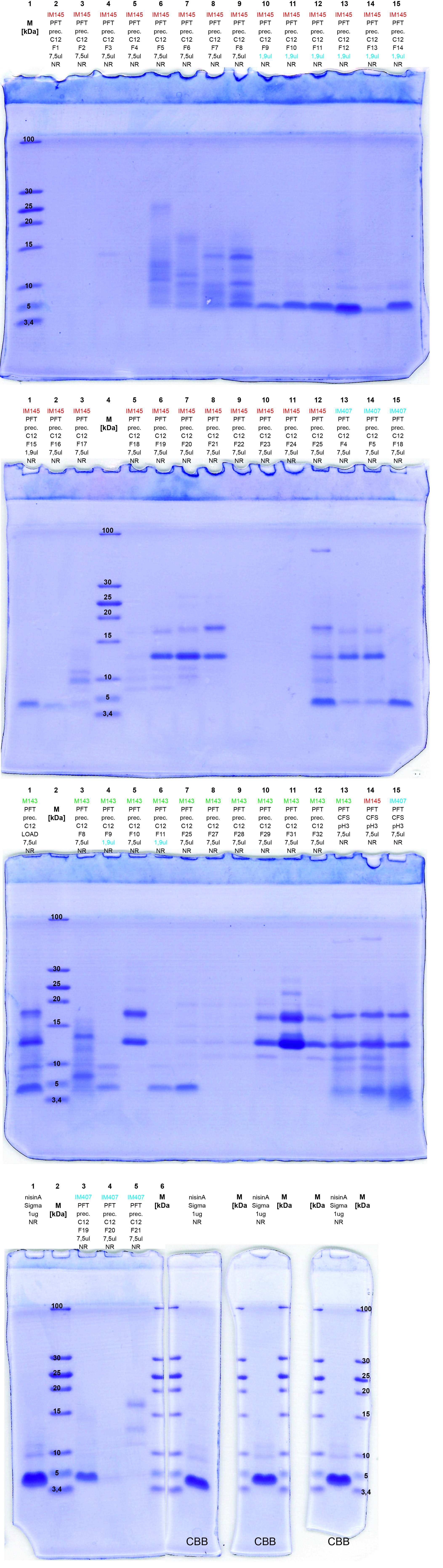

Supplement: Supplementary file 2 — Supplementary Material 2. [file 12866_2025_4543_MOESM2_ESM.jpg]
